# Analysis of Cross-Reactive Antibodies Recognizing the Fusion Loop of Envelope Protein and Correlation with Neutralizing Antibody Titers in Nicaraguan Dengue Cases

**DOI:** 10.1371/journal.pntd.0002451

**Published:** 2013-09-19

**Authors:** Chih-Yun Lai, Katherine L. Williams, Yi-Chieh Wu, Sarah Knight, Angel Balmaseda, Eva Harris, Wei-Kung Wang

**Affiliations:** 1 Department of Tropical Medicine, Medical Microbiology and Pharmacology, John A. Burns School of Medicine, University of Hawaii at Manoa, Honolulu, Hawaii, United States of America; 2 Division of Infectious Diseases and Vaccinology, School of Public Health, University of California, Berkeley, Berkeley, California, United States of America; 3 National Virology Laboratory, National Center for Diagnosis and Reference, Ministry of Health, Managua, Nicaragua; Florida Gulf Coast University, United States of America

## Abstract

Dengue virus (DENV) is the leading cause of arboviral diseases in humans worldwide. The envelope (E) protein of DENV is the major target of neutralizing antibodies (Abs). Previous studies have shown that a significant proportion of anti-E Abs in human serum after DENV infection recognize the highly conserved fusion loop (FL) of E protein. The role of anti-FL Abs in protection against subsequent DENV infection versus pathogenesis remains unclear. A human anti-E monoclonal Ab was used as a standard in a virion-capture ELISA to measure the concentration of anti-E Abs, [anti-E Abs], in dengue-immune sera from Nicaraguan patients collected 3, 6, 12 and 18 months post-infection. The proportion of anti-FL Abs was determined by capture ELISA using virus-like particles containing mutations in FL, and the concentration of anti-FL Abs, [anti-FL Abs], was calculated. Neutralization titers (NT_50_) were determined using a previously described flow cytometry-based assay. Analysis of sequential samples from 10 dengue patients revealed [anti-E Abs] and [anti-FL Abs] were higher in secondary than in primary DENV infections. While [anti-FL Abs] did not correlate with NT_50_ against the current infecting serotype, it correlated with NT_50_ against the serotypes to which patients had likely not yet been exposed (“non-exposed” serotypes) in 14 secondary DENV3 and 15 secondary DENV2 cases. These findings demonstrate the kinetics of anti-FL Abs and provide evidence that anti-FL Abs play a protective role against “non-exposed” serotypes after secondary DENV infection.

## Author Summary

The four serotypes of dengue virus (DENV) are the leading cause of mosquito-borne viral diseases in humans. Whereas infection with one DENV serotype is thought to confer protection against re-infection with that serotype, it can be either protective or enhance disease severity upon subsequent (“secondary”) infection with a different serotype. The envelope (E) protein of DENV is the major target of neutralizing antibodies. Previously, we and others reported that a significant proportion of anti-E antibodies in human dengue-immune sera recognize the fusion loop (FL) of E protein. The role of anti-FL antibodies in protection against subsequent DENV infections versus pathogenesis remains unclear. In this study, we developed capture ELISAs to measure the concentrations of anti-E and anti-FL antibodies in sera of Nicaraguan dengue patients collected 3, 6, 12 and 18 months post-illness, and investigated the kinetics of these antibodies and their relationship to neutralization activity. While the concentrations of anti-FL antibodies did not correlate with 50% neutralization titers (NT_50_) against the current infecting serotype, it correlated with NT_50_ against serotypes to which patients had likely not yet been exposed (“non-exposed” serotypes) in secondary DENV infections. These findings provide evidence that anti-FL antibodies play a protective role against “non-exposed” serotypes after secondary DENV infection.

## Introduction

The four serotypes of dengue virus (DENV1–4) are the leading cause of arboviral diseases in humans in tropical and subtropical regions [1], [2]. It has been estimated that more than 3 billion people in over 100 countries are at risk of infection and 50–100 million DENV infections occur annually worldwide [1], [2]. The clinical presentation after DENV infection ranges from asymptomatic infection to a self-limited illness, dengue fever (DF), to severe and potentially life-threatening disease, dengue hemorrhagic fever/dengue shock syndrome (DHF/DSS) [1], [2]. While considerable efforts have been made to develop interventions, currently there is no licensed vaccine or antiviral therapeutic against dengue available [3].

DENV belongs to the genus *Flavivirus* in the family *Flaviviridae*. It contains a positive-sense RNA genome of approximately 11 kilobases in length. Flanked by the 5′ and 3′ untranslated regions, the genome has a single open reading frame encoding a polyprotein precursor, which is cleaved by cellular and viral proteases into three structural proteins, the capsid, precursor membrane (prM) and envelope (E), and seven non-structural proteins [4]. The E protein forms 90 “head-to-tail” homodimers on the surface of mature virions [4]–[6]. The E protein participates in virus entry and is the major target of neutralizing antibodies (Abs) [3], [4]. In the presence of non-neutralizing or suboptimal concentrations of neutralizing anti-E Abs, DENV replicates to higher titers in human Fcγ receptor-bearing cells *in vitro*, a phenomenon known as antibody-dependent enhancement (ADE) [7]–[9]. The ectodomain of E protein contains three domains [10]. Domain II contains an internal fusion loop (FL) that is involved in membrane fusion. Domain III is believed to participate in receptor binding [4], [10], [11].

In the genus *Flavivirus*, there are several serocomplexes, including DENV, Japanese-encephalitis virus, and tick-borne encephalitis virus serocomplexes. Abs that recognize members from different serocomplexes, members within a serocomplex, or a single member are called flavivirus group-reactive, complex-reactive, or type-specific, respectively [12]. Previous studies of polyclonal human sera revealed that a significant proportion of anti-E Abs after DENV infection was group-reactive and recognized the FL of domain II, whereas only a minor proportion was type-specific and recognized E domain III [13]–[16]. The change in the amount of anti-FL Abs over time and the role of anti-FL Abs in dengue protection versus pathogenesis remain unclear. Following primary DENV infection, individuals develop monotypic neutralizing Abs against the infecting serotype [9]. A recent study on depletion of human sera by recombinant E protein has shown that cross-reactive Abs (including anti-FL Abs) do not contribute substantially to monotypic neutralization against the infecting serotype after primary DENV infection [17]. After secondary DENV infection, individuals develop not only neutralizing Abs against serotypes to which they have been previously exposed but also cross-reactive neutralizing Abs against serotypes to which they have not yet been exposed (“non-exposed” serotypes) [9]. The nature of such heterotypic neutralizing Abs remains unknown. We hypothesize that the cross-reactive anti-FL Abs may play a role in protection against the non-exposed serotypes after secondary infection.

In this study, we developed a DENV virion-capture ELISA to measure the concentrations of anti-E Abs, [anti-E Abs], against DENV in sera, determined the proportion of anti-FL Abs (% anti-FL Abs) by a previously-described capture ELISA using virus-like particles (VLPs) [14], [16], and calculated the concentrations of anti-FL Abs, [anti-FL Abs]. We examined the changes of [anti-E Abs] and [anti-FL Abs] over time in sequential serum samples from 10 cases of primary or secondary DENV infection and then measured the [anti-FL Abs] in 26 additional secondary DENV infections. While [anti-FL Abs] did not correlate with NT_50_ against the current infecting serotype, it correlated with neutralization titers against likely “non-exposed” serotypes in 29 secondary infections. These findings support our hypothesis that anti-FL Abs may play a protective role against the “non-exposed” serotypes after secondary DENV infection.

## Materials and Methods

### Ethics statement and human sera

Thirty-six laboratory-confirmed dengue patients, who were admitted to the Hospital Infantil Manuel de Jesús Rivera in Managua, Nicaragua between October 2006 and October 2008 and followed up for 18 months, were selected arbitrarily for the analysis. The study was approved by the Institutional Review Boards of the University of California, Berkeley, and the Nicaraguan Ministry of Health. Parents or legal guardians of all subjects provided written informed consent, and subjects 6 years of age and older provided assent. DENV infection was confirmed by detection of viral RNA by RT-PCR directed to the capsid region [18], [19]; virus isolation in C6/36 cells [20]; IgM seroconversion between acute and convalescent-phase samples; and/or a ≥4-fold increase in total anti-DENV Abs between acute and convalescent-phase samples as measured by Inhibition ELISA [19], [21]. Primary DENV infection was defined by an Ab titer by Inhibition ELISA [21] of <10 in acute samples or <2,560 in convalescent (day 14) samples, while secondary infection was defined by an Ab titer by Inhibition ELISA of ≥10 in acute samples or ≥2,560 in convalescent samples [22]. Disease severity was classified according to the 1997 World Health Organization Guidelines [23].

### Proportion of anti-FL Abs determined by capture ELISA using wild type (WT) and mutant VLPs

The plasmids expressing prM/E proteins of DENV1 (Hawaii strain, pCB-D1) and DENV2 (strain 16681) were used to generate WT and mutant VLPs containing mutations at critical FL residues (W101A plus F108A) as described previously [16], [24]. VLPs derived from ultracentrifugation of culture supernatants of transfectants of 293T cells were used in a capture ELISA as described previously [14], [16]. Briefly, 96-well plates were coated with rabbit anti-serum against DENV1 at 4°C overnight, followed by blocking with 1% BSA in 1× PBS for 1 hour. VLPs and mutant VLPs (at ∼0.01 µg/ml) were added, followed by two-fold serial dilutions (using dilution buffer) of each human serum sample, anti-human IgG conjugated to horseradish-peroxidase (HRP), TMB substrate and stop solution [16]. The absorbance at a wavelength of 450 nm (OD 450) with reference wavelength of 650 nm was read. The endpoint titers were calculated as the reciprocal of the highest titers that yielded a signal greater than 3 standard deviations of the mean signal from multiple (n = 5) normal human sera. The proportion of anti-FL Abs was determined by the formula: % anti-FL Abs = [1 - endpoint titer to mutant VLPs/endpoint titer to WT VLPs]×100% [14]. Mixtures of mAbs containing different proportions of a mouse anti-FL mAb (FL0231) and a mouse anti-E domain III mAb (DA6-7) in the VLP-capture ELISA revealed a linear relationship between the proportion of FL0231 (anti-FL mAb) added and the measured proportion of FL0231 (*P* = 0.003, two-tailed Spearman correlation test) (Figure S1). The limit of detection was 4% based on the experiment of mixing mAbs (Figure S1).

### Concentration of anti-E Abs determined by virion-capture ELISA

**Figure 1:**
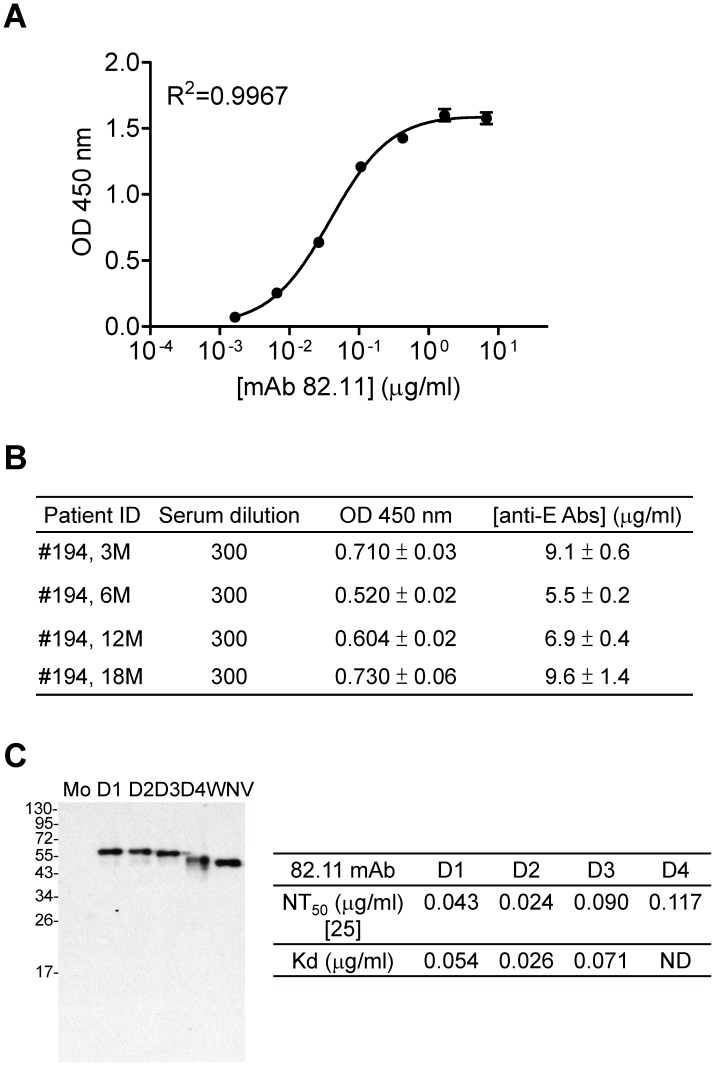
Measurement of [anti-E Abs] in human sera by capture ELISA. (A) Virion-capture ELISA was performed using serial dilutions of a human mAb 82.11 with known **c**oncentrations to generate a standard curve. (B) Sequential human serum samples from a dengue patient were tested simultaneously with the standard and the OD values were interpolated to determine [anti-E Abs]. Data are means with standard deviation of duplicates from one representative experiment of two. (C) The binding specificity of mAb 82.11 was determined by Western blot analysis using cell lysates collected from Vero cells infected with mock, DENV1 (Hawaii strain), DENV2 (NGC strain), DENV3 (H87 strain), DENV4 (H241 strain) or WNV (NY99 strain) [16]. The NT_50_ [25] and dissociation constant (Kd) of mAb 82.11 are summarized on the right. The viron-capture ELISA of DENV1, DENV2 and DENV3 was performed using serial dilutions of mAb 82.11; the Kd was determined using the program GraphPad Prism 5.0. Data are means of duplicates from one representative experiment of two. ND, not done.

The virion-capture ELISA was performed similarly to the VLP-capture ELISA except that DENV1 (Hawaii strain) virions derived from ultracentrifugation of culture supernatants of infected Vero cells were used as antigen. Briefly, 96-well plates were coated with rabbit anti-serum against DENV1 at 4°C overnight, followed by blocking with 1% BSA in 1× PBS for 1 hour and adding DENV1 virions (at ∼0.01 µg/ml). To determine the [anti-E Abs], the virion capture-ELISA was initially performed using two-fold serial dilutions of each serum to identify the dilution that gave rise to OD values within the linear range of the standard curve (from 6.6 to 105.63 ng/ml), based on known concentrations of a human mAb 82.11 that targets the FL epitope (Figure 1A) [25]. Then the virion capture-ELISA was performed using such serum dilution in parallel to mAb 82.11 (from 1.65 to 6760 ng/ml) in duplicates. The OD values were interpolated to determine the [anti-E Abs] in each human serum sample (Figure 1B) (GraphPad Prism 5.0, GraphPad software Inc., CA).

### Neutralization test

The 50% neutralization titer (NT_50_) was determined using a flow cytometry-based neutralization assay with reporter viral particles (RVPs) of four different DENV serotypes as described previously [26]. Briefly, eight 3-fold dilutions of each serum sample were mixed with DENV RVPs (a GFP DENV reporter replicon packaged by DENV structural proteins C-prM/M-E expressed *in trans*) [26], [27] and incubated with human Raji-DC-SIGNR cells for 48 hours. GFP-positive infected cells were quantified by flow cytometry, and raw data extracted using FlowJo software, version 7.2.5 (TreeStar Software) was graphed in the GraphPad Prism 5.0 program as percent infection versus the log of the reciprocal serum dilution. A sigmoidal dose response curve with a variable slope was then generated to determine NT_50_, the Ab dilution at which a 50% reduction in infection was observed compared to the no-Ab control [26], [28]. For patients with secondary DENV infection, the current infecting serotype was identified by RT-PCR and/or virus isolation, and the previous infecting serotype(s) were determined based on the neutralization pattern, the epidemiology of dominant DENV serotype circulation in Nicaragua, and the age of the patients [29]. For each patient, the remaining serotypes were considered as likely “non-exposed serotypes”.

### Statistical analysis

The two-tailed Mann-Whitney test was used to determine the difference in [anti-FL Abs] and [anti-E Abs] among primary and secondary DENV infections. The one-tailed Spearman correlation test was used to determine the relationship between [anti-FL Abs] and NT_50_ using the program GraphPad Prism 5.0. The two-tailed Spearman correlation test was used to determine the relationship between the proportion of anti-FL mAb FL0231 added and that measured in the VLP-ELISA.

## Results

### Determination of [anti-E Abs], [anti-FL Abs] and % anti-FL Abs in human sera

We first developed a virion-capture ELISA using known concentrations of a human anti-E mAb, 82.11, to generate a standard curve (Figure 1A) [25]; the OD values derived from human sera in the same capture ELISA were interpolated to determine [anti-E Abs] (Figure 1B). MAb 82.11 bound to the E protein of the four DENV serotypes equivalently, as shown by Western blot analysis and virion-capture ELISA (Figure 1C), which is in agreement with previous reports of its neutralization potency against the four DENV serotypes at comparable concentration [25]. Table 1 summarizes the [anti-E Abs] in sera collected longitudinally 3, 6, 12 and 18 months post-illness from 6 confirmed dengue cases with primary DENV infection and 4 dengue cases with secondary infection. The [anti-E Abs] ranged from 5.8 to 158.8 µg/ml and 58.8 to 1894.9 µg/ml in primary and secondary DENV infections, respectively.

**Table 1:** Concentration of anti-E Abs and anti-FL Abs and proportion of anti-FL Abs in sequential serum samples from 10 dengue cases.

Patient ID	Immune status^a^	Current infecting serotype^b^	Sampling time (month)^c^	[anti-E Abs] (µg/ml)^d^	% anti-FL Abs (%)^d^	[anti-FL Abs] (µg/ml)^d^
256	primary	D1	3	158.8±37.9	41±4	65.1
			6	118.4±6.6	49±5	58.0
			12	66.8±2.9	25±0	16.7
312	primary	D1	3	86.0±6.7	32±2	27.5
			12	64.2±0.4	BD^e^	BD^e^
173	primary	D2	3	6.3±0.8	33±2	2.1
			6	6.6±0.9	27±4	1.8
			12	9.1±0.5	27±4	2.5
			18	10.4±1.0	20±3	2.1
194	primary	D2	3	9.1±0.6	10±9	0.9
			6	5.5±0.2	18±7	1.0
			12	6.9±0.4	24±5	1.7
			18	9.6±1.4	32±6	3.1
208	primary	D3	3	57.9±0.4	63±2	36.5
			6	41.4±1.2	45±7	18.6
			12	34.2±0.1	41±5	14.0
			18	38.2±5.1	34±3	13.0
265	primary	D3	3	36.0±0.3	33±2	11.9
			6	26.1±0.9	16±6	4.2
			12	33.3±2.1	BD^e^	BD^e^
			18	52.5±2.5	BD^e^	BD^e^
274	secondary	D1	3	188.7±5.9	31±3	58.5
			6	781.3±34.2	61±5	476.6
			12	58.8±0.5	29±3	17.1
			18	62.3±1.4	14±2	8.7
233	secondary	D2	3	529.1±124.8	31±3	164.0
			6	178.1±8.6	22±2	39.2
			12	85.7±5.4	24±5	20.6
			18	69.6±2.1	26±4	18.1
237	secondary	D2	3	1894.9±145.5	57±2	1080.1
			6	1472.5±354.6	59±5	868.8
			12	952.0±111.8	56±4	533.1
			18	851.4±106.4	58±5	493.8
240	secondary	D2	3	721.2±179.0	20±4	144.2
			12	360.8±128.0	20±9	72.2
			18	333.2±27.2	15±4	50.0

a Primary or secondary DENV infection was determined as described in Methods.

b The current infecting serotype was determined as described in Methods. With the exception of two DHF/DSS cases (274, 233), all others were DF cases. D = DENV.

c Sampling time was determined relative to onset of fever.

d [anti-E Abs], [anti-FL Abs] and % anti-FL Abs were determined as described in Methods.

e Below the limit of detection (BD). The limit of detection of % anti-FL Abs is 4% as described in Methods.

**Figure 2:**
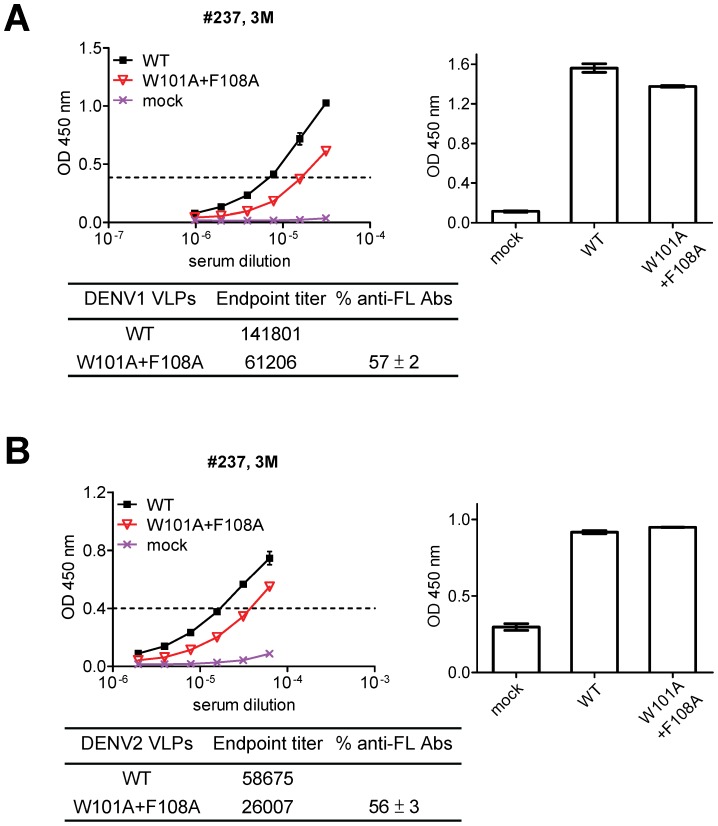
Determination of % anti-FL Abs in serum of a dengue patient by VLP-capture ELISA. (A) Serial dilutions of the serum were subjected to a capture ELISA using DENV1 WT and mutant VLPs containing mutations in the FL epitope (W101A+F108A). The bar graph displaying results from an anti-E ELISA shows that comparable amounts of WT and mutant VLPs were added based on recognition of E by pooled human dengue-immune sera. % anti-FL Abs = [1 – endpoint titer to mutant VLPs/endpoint titer to WT VLPs]×100%. (B) The same serum was subjected to a capture ELISA using DENV2 WT and mutant VLPs (W101A+F108A). Data are presented as in (A). Data are means with standard deviation of duplicates from one representative experiment of two. For endpoint titers, only means are shown.

We next employed a previously-described VLP-capture ELISA using DENV1 WT and mutant VLPs containing representative FL mutations (W101A plus F108A) to determine the % anti-FL Abs in each serum sample [14], [16]. As shown in Figure 2A, the % anti-FL Abs in the 3-month post-illness serum of a secondary DENV2 case (ID 237) was 57%. We also examined the same serum by using DENV2 WT and mutant VLPs (W101A plus F108A) in the VLP-capture ELISA, and the % anti-FL Abs was found to be 56%. The % anti-FL Abs determined by DENV1 and DENV2 VLP-capture ELISA was similar in the 6-month and 18-month sera of ID237 and in the sera of other cases as well (Figure S2). This finding is consistent with the notion that the FL residues recognized by anti-FL Abs are highly conserved by different flaviviruses, and therefore the % anti-FL Abs determined by VLPs of different DENV serotypes was similar. We thus used DENV1 VLP-capture ELISA to determine the % anti-FL Abs for all sera in this study. Table 1 summarizes the % anti-FL Abs in sequential serum samples from 10 cases, which ranged from 4 to 63%. After determining the % anti-FL Abs we calculated the [anti-FL Abs], which ranged from 0 to 0.94 µg/ml (Table 1).

### Kinetics of [anti-E Abs] and [anti-FL Abs] over time

**Figure 3:**
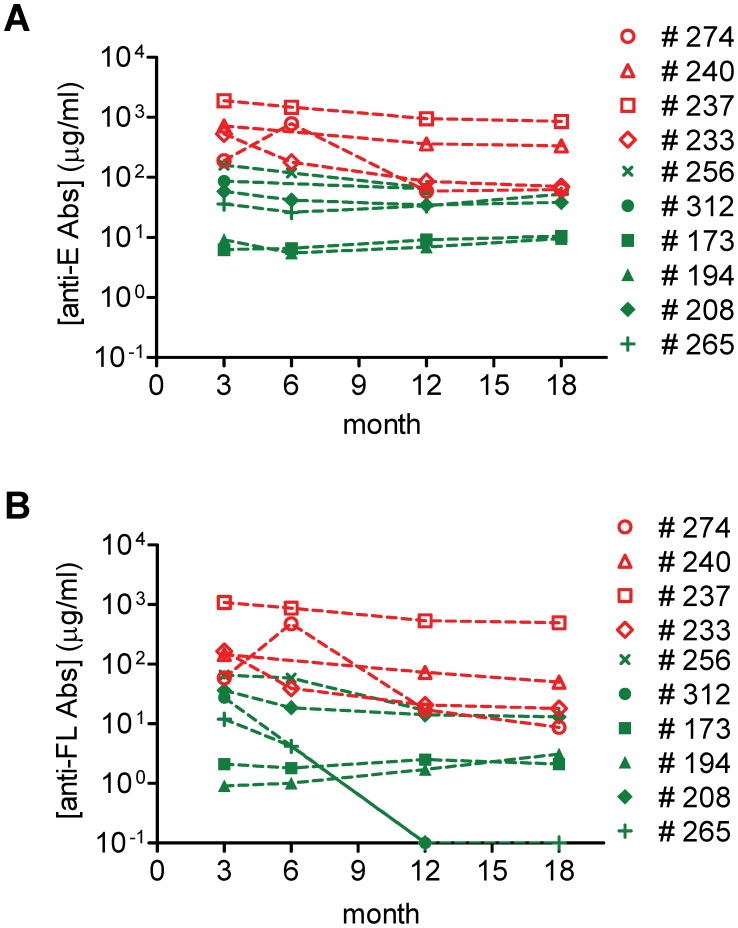
Kinetics of [anti-E Abs] and [anti-FL Abs] in sera of dengue patients. (A) [anti-E Abs] and (B) [anti-FL Abs] in samples collected 3, 6, 12 and 18 months post-infection. Green closed symbols, patients with primary DENV infection; red open symbols, patients with secondary DENV infection.

As shown in Figure 3A, the [anti-E Abs] in most cases slightly decreased from 3 months to 6 months after infection except one DHF case, ID 274, that displayed peak anti-E Abs 6 months after infection. There was no difference in [anti-E Abs] between 6 and 12 months and between 12 and 18 months among primary DENV infections (*P* = 0.66 and 1, respectively) and secondary infections (*P* = 0.4 and 0.88, respectively, two-tailed Mann-Whitney test). The [anti-E Abs] was higher in secondary infections than in primary infections (*P* = 0.02, 0.07, 0.01 and 0.06, at 3, 6, 12 and 18 months, respectively, two-tailed Mann-Whitney test). A similar trend in the [anti-FL Abs] was noted, again except ID 274, where [anti-FL Abs] peaked 6 months after infection (Figure 3B). The [anti-FL Abs] was also significantly higher in secondary as compared to primary DENV infections (*P* = 0.02, 0.04, 0.04 and 0.03, at 3, 6, 12 and 18 months, respectively, two-tailed Mann-Whitney test). Notably, two cases, ID312 (primary DENV1 infection) and ID265 (primary DENV3 infection), had undetectable anti-FL Abs 12 months after infection.

### Relationship between anti-FL Abs and neutralizing Abs in secondary DENV infections

We then examined the [anti-E Abs], % anti-FL Abs and [anti-FL Abs] in sera collected 12 months post-illness from another 26 patients with secondary DENV infection (Table S1). Of the total of 30 secondary DENV infections, 13 were classified as DF and 17 as DHF/DSS. No significant difference was observed in the [anti-E Abs], % anti-FL Abs or [anti-FL Abs] at 12 months post-infection between DF and DHF/DSS patients (*P* = 0.48, 0.79, and 0.43, respectively, two-tailed Mann-Whitney test), although the number of cases examined was small.

We next measured neutralizing Abs in each serum sample against the four DENV serotypes using a previously described flow cytometry-based assay with DENV RVPs [26]. The NT_50_ in 12-month sera from the 30 secondary DENV infections (15 DENV2, 14 DENV3 and 1 DENV1) are summarized in Table 2. For each patient, the previous infecting serotypes were determined based on the neutralization pattern, the epidemiological history of dominant DENV serotype circulation in Nicaragua, and the age of the patient [29]. The serotypes excluding the current and previous infecting serotype(s) were considered as likely “non-exposed serotypes”.

**Table 2:** Neutralization titers in sera of 30 secondary dengue cases 12 months post-infection.

Patient ID^a^	Disease severity^b^	Age (year born)	Current infecting serotype^c^	Previous infecting serotype(s)^d^	NT_50_ e				[anti-FL Abs] (µg/ml)^f^
					DENV1	DENV2	DENV3	DENV4	
133	DF	11 (1995)	D2	D4	133	142	32	*905	68.1
233	DHF	3 (2004)	D2	D1	*460	116	96	186	20.6
237	DF	14 (1993)	D2	D1	*1454	223	764	812	533.1
240	DF	5 (2002)	D2	D1	*2421	732	639	307	72.2
274	DSS	14 (1993)	D1	D3	287	82	*1311	165	17.1
295	DHF	9 (1998)	D2	D4	211	1453	53	*971	44.0
296	DSS	14 (1993)	D2	D1	*2196	524	183	244	58.8
299	DSS	11 (1996)	D2	D3	839	531	*1452	311	211.4
300	DF	13 (1994)	D2	D1 and/or D3	*1666	979	*1870	191	26.2
301	DSS	13 (1994)	D2	D3	168	1444	*743	87	151.3
303	DHF	9 (1998)	D2	D3	276	195	*1995	560	73.5
305	DHF	14 (1993)	D2	-g	413	527	118	317	80.7
306	DHF	13 (1994)	D2	D1	*521	217	151	75	14.2
309	DF	14 (1993)	D2	D1	*2090	1265	330	322	114.0
316	DF	13 (1994)	D2	D1	*1134	558	166	332	84.9
341	DHF	8 (2000)	D2	D1	*1092	735	203	327	56.0
351	DHF	13 (1995)	D3	-g	136	111	128	56	40.1
368	DSS	2 (2006)	D3	-g	687	373	478	282	305.4
374	DHF	3 (2005)	D3	D2	316	*527	244	48	21.1
385	DF	8 (2000)	D3	-g	225	48	295	155	49.3
395	DF	7 (2001)	D3	D1	*719	49	122	57	63.7
400	DHF	10 (1998)	D3	-g	84	152	217	67	130.8
403	DF	7 (2001)	D3	-g	306	432	800	56	60.9
412	DF	9 (1999)	D3	D1	*1971	126	432	119	70.5
421	DF	9 (1999)	D3	D2	139	*313	115	32	19.5
429	DSS	6 (2002)	D3	D1	*1102	27	156	31	55.4
433	DF	13 (1995)	D3	D1	*504	178	170	173	216.8
444	DHF	1 (2007)	D3	D2	630	*5977	372	128	10.2
452	DHF	7 (2001)	D3	D1	*589	56	153	56	31.8
454	DF	10 (1998)	D3	D2	66	*216	63	51	2.9

a , Secondary DENV infection was determined as described in Methods.

b DF, Dengue Fever; DHF, Dengue Hemorrhagic Fever; DSS, Dengue Shock Syndrome.

c Current infecting serotype was determined by RT-PCR and/or virus isolation of acute samples. D = DENV.

d Previous infecting serotypes were determined based on the neutralization pattern, the epidemiology of dominant DENV serotype circulation in Nicaragua, and the age of patients [29]. Asterisks indicate the highest NT_50_, which suggests the previous infecting serotypes consistent with the epidemiological history.

e NT_50_ was determined as described in Methods.

f [anti-FL Abs] was determined as described in Methods.

g Previous infecting serotype(s) could not be determined.

**Figure 4:**
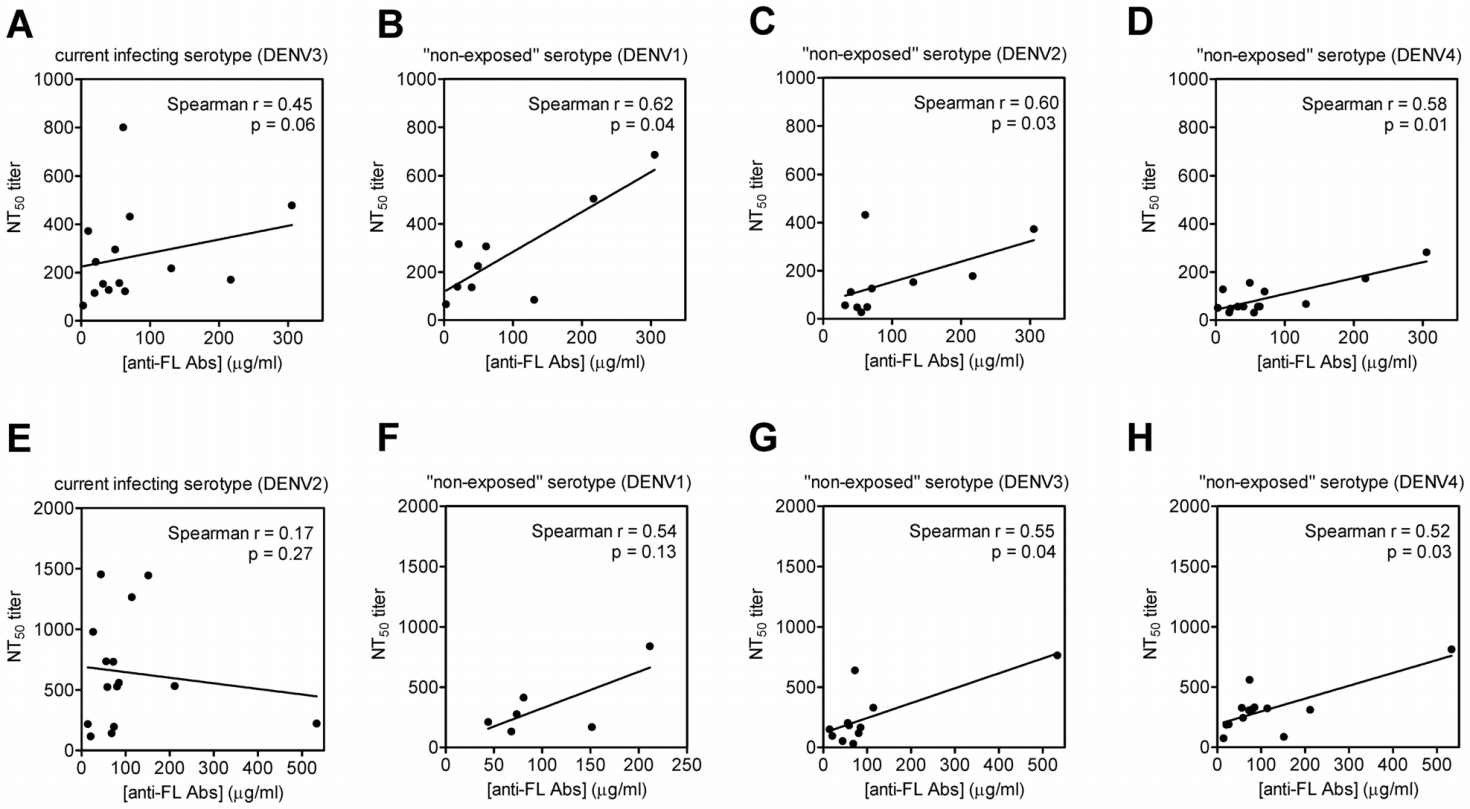
Relationship between [anti-FL Abs] and NT_50_ against current infecting and “non-exposed” serotypes in secondary DENV infection. The current infecting serotype, previous infecting serotype(s) and “non-exposed serotypes” were determined as described in Methods. [anti-FL Abs] and NT_50_ against the current infecting serotype (A, E) and “non-exposed” serotypes (B to D, F to H) in patients with secondary DENV3 (A to D) and secondary DENV2 (E to H) infections.

We then examined the relationship between [anti-FL Abs] and NT_50_ 12 months post-infection in patients with secondary DENV infection. [anti-FL Abs] did not correlate with the NT_50_ against the current infecting serotype (Figures 4A and 4E). However, [anti-FL Abs] did correlate with the NT_50_ against the likely “non-exposed” serotypes (DENV4 and DENV2 and DENV1) for patients with secondary DENV3 infection (*P* = 0.01, *P* = 0.03 and *P* = 0.04, respectively, one-tailed Spearman correlation test) (Figures 4B to 4D). For patients with secondary DENV2 infection, [anti-FL Abs] correlated with the NT_50_ against the “non-exposed” serotypes (DENV4 and DENV3) but not DENV1, likely due to the small sample size of this subgroup (*P* = 0.03, *P* = 0.04 and *P* = 0.13, respectively, one-tailed Spearman correlation test) (Figures 4F to 4H).

## Discussion

We and others have previously reported that a significant proportion of anti-E Abs in human dengue-immune sera recognize the highly conserved FL residues in domain II of E protein, whereas a small proportion of anti-E Abs recognize E domain III residues [13]–[16]. The role of anti-FL Abs in protection against subsequent infections and/or dengue pathogenesis remains unclear. In this study, we first developed capture ELISAs to measure the [anti-E Abs] and [anti-FL Abs] in sera from dengue patients to investigate the kinetics of anti-E Abs and anti-FL Abs over time and found that [anti-E Abs] and [anti-FL Abs] were quite stable across time and were higher in secondary DENV infections than in primary infections. We then examined the relationship between [anti-FL Abs] and NT_50_ in patients with secondary DENV infection. While [anti-FL Abs] did not correlate with NT_50_ against the current infecting serotype, it correlated with NT_50_ against likely “non-exposed” serotypes (DENV4, DENV2 and DENV1) in 14 secondary DENV3 cases and “non-exposed” serotypes (DENV4 and DENV3) in 15 secondary DENV2 cases. These findings suggest that anti-FL Abs may play a protective role against “non-exposed” serotypes after secondary DENV infection.

It is known that after primary DENV infection, individuals develop life-long protection against the infecting serotype, which correlates with the appearance of monotypic neutralizing Abs against the infecting serotype [30]–[34]. The type-specific anti-E neutralizing Abs rather than the group-reactive anti-FL Abs generated after primary infection are believed to contribute to such monotypic neutralizing activity. This concept was supported by the observation that the monotypic neutralizing activity in human sera after primary infection was greatly reduced by depleting type-specific binding activity with virions of the infecting serotype but was not substantially reduced by depleting cross-reactive binding activity (including anti-FL Abs) with virions of other serotypes [17]. Consistent with this, we found that [anti-FL Abs] did not correlate with NT_50_ against the infecting serotype in patients with primary DENV infection (data not shown). The nature of the type-specific anti-E neutralizing Abs, which were initially thought to be those targeting E domain III based on the studies of anti-domain III mAbs [35]–[38], were recently reported to be those targeting quaternary epitopes spanning adjacent E dimers on the virion or possibly other as yet to be identified epitopes [17], [39].

After secondary DENV infection, individuals develop not only neutralizing Abs against serotypes to which they have been previously exposed but also heterotypic neutralizing Abs against serotypes to which they have not yet been exposed [9]. The heterotypic neutralizing Abs are believed to account for heterotypic protection against subsequent infection by non-experienced serotypes and contribute to the very low numbers of hospital admission observed after a third or fourth DENV infection in humans [40] as well as the low rate of viremia after a third DENV infection in monkeys [41]–[44]. The nature of such heterotypic neutralizing Abs remains unknown. One possibility is that some complex-reactive anti-E Abs contribute to the neutralizing activities against “non-exposed” serotypes after secondary infection. Alternatively, the group-reactive anti-FL Abs generated after secondary DENV infection may contribute to such neutralizing activity. Our findings that [anti-FL Abs] correlated with NT_50_ against likely “non-exposed serotypes” in secondary cases suggest that anti-FL Abs contribute significantly to heterotypic neutralizing activity against “non-exposed” serotypes after secondary DENV infection. Nonetheless, it is likely that such heterotypic neutralizing Abs against “non-exposed” serotypes include other non-FL cross-reactive Abs as well.

The epitopes recognized by anti-FL Abs include several key residues such as 101W, 106G, 107L and 108F in the FL of E domain II [16], [45], which are highly conserved by different flaviviruses and absolutely conserved by the four DENV serotypes. It is conceivable that during secondary DENV infection, memory B cells recognizing these highly conserved FL residues expand and generate anti-FL Abs with higher avidity through affinity maturation [46]. Consistent with this, studies of human sera after DENV infection, which likely contained a significant proportion of anti-FL Abs, showed that the binding avidity of anti-DENV Abs from secondary infections was higher than that from primary infections [28], [47]. Moreover, it was reported recently that cross-reactive memory B cells or plasma cells as well as serum avidity increase greatly during acute secondary DENV infection, with greater reactivity to the previous infecting serotype than the current infecting serotype [28], [48]. Future studies involving experiments that remove cross-reactive anti-FL Abs from the sera of secondary DENV infections and examine the neutralizing activity against “non-exposed” serotypes will help to further elucidate the contribution of anti-FL Abs to heterotypic neutralizing activity. It is worth noting that while anti-FL Abs are cross-reactive to all four DENV serotypes, [anti-FL Abs] did not correlate with NT_50_ against the current infecting serotype (Figures 4A and 4E). This suggests that type-specific or other non-FL Abs probably dominate the neutralizing activity against the current infecting serotype.

In this study, we used known concentrations of a human anti-E mAb (82.11) as a standard in our quantitative virion-capture ELISA to measure [anti-E Abs] in human sera. MAb 82.11, which recognizes FL residues and is a group-reactive neutralizing Ab against four DENV serotypes [25, data not shown], was used as a reference for anti-E Abs in human sera because group-reactive anti-FL Abs constitute a significant proportion of anti-E Abs in human serum. The possibility that some anti-E Abs had different binding properties to the DENV virion compared with 82.11 and were thus over- or under-estimated in the quantification cannot be completely ruled out. To further validate the use of DENV1 virion- and VLP-capture in determining the [anti-E Abs], % anti-FL Abs and [anti-FL Abs] in this study, we used DENV3′ virion- and VLP-capture ELISA systems to determine the [anti-E Abs], % anti-FL Abs and [anti-FL Abs] in 14 DENV3 infection samples, and found a nice correlation with these three values determined by the two systems (DENV1 vs. DENV3: r = 0.91, *P*<0.0001; r = 0.78, *P* = 0.0009; and r = 0.74, *P* = 0.0027, respectively, two-tailed Spearman correlation test) (Figure S3). In addition, we determined the [anti-E Abs], % anti-FL Abs and [anti-FL Abs] of 7 DENV2 infection samples using DENV2 virion- and VLP-capture ELISA systems, yielding a good correlation (DENV1 vs. DENV2: r = 0.9, *P* = 0.0046; r = 0.96, *P* = 0.0028; and r = 1, *P* = 0.0004, respectively, two-tailed Spearman correlation test) (Figure S3).

It is also worth noting that the [anti-E Abs] and [anti-FL Abs] determined were for IgG. To address the possibility that IgM might confound these values, we used a previously described IgM ELISA [20], [21] to measure anti-DENV IgM and found IgM was negative for all 36 serum samples at 12 months (data not shown), suggesting that IgM is unlikely to confound the 12-month [anti-FL Abs] determined and thus the correlation with NT_50_ analyzed in Figure 4. Another concern is that anti-prM Abs might affect the anti-E Abs determined. Previous studies have shown that the level of anti-prM Abs in dengue-immune sera (either primary or secondary DENV infection) was much lower than that of anti-E Abs based on the intensity of prM and E bands in Western blot analysis, where the antigens were derived from virus-infected cell lysates and presumably contained equal molar ratios of prM and E proteins [13], [49]–[51]. We used serial dilutions of known concentrations of anti-prM and anti-E human mAbs together with human serum on the same blot and estimated the level of anti-prM Abs in human serum to be 30 fold less than anti-E Abs (data not shown). Therefore, the amount of anti-prM Abs detected in our virion-capture ELISA is trivial compared with that of anti-E Abs, though the possibility of confounding cannot be completely ruled out. Nonetheless, the [anti-E Abs] thus measured, demonstrating higher concentrations in secondary DENV infections as compared to primary infections, is in agreement with previous reports using other methods such as the plaque reduction neutralization test and endpoint dilution determined by ELISA [9], [15], [52]. Moreover, the [anti-FL Abs] calculated based on [anti-E Abs] and % anti-FL Abs showed a correlation with NT_50_ against likely “non-exposed” serotypes in secondary DENV infections, which is consistent with the historical observations of heterotypic neutralization against “non-exposed” serotypes after secondary DENV infection [9]. In summary, our assay for [anti-FL Abs] provides a simple and quantitative method to study the role of anti-FL Abs in protection against or enhancement of dengue disease.

## Supporting Information

Figure S1 **Determination of % anti-FL Abs in mixtures of mAbs containing different proportions of an anti-FL mAb by VLP-capture ELISA.** (A) Increasing amounts of mouse mAb FL0231, which recognizes the FL, was mixed with mouse mAb DA6-7, which recognizes E domain III, such that the proportion of anti-FL mAb increased from 0% to 100%. Different mixtures were subjected to a capture ELISA using DENV1 WT and mutant VLPs containing mutations in the FL epitope (W101A+F108A). The bar graph displaying results of an anti-E ELISA shows that comparable amounts of WT and mutant VLPs were added based on recognition of E by pooled human dengue-immune sera. (B) A linear relationship between the proportion of FL0231 (anti-FL mAb) added and the measured proportion of FL0231 was noted (*P* = 0.003, two-tailed Spearman correlation test). % anti-FL Abs (measured proportion of FL0231) = [1 – endpoint titer to mutant VLPs/endpoint titer to WT VLPs]×100%. Data are means with standard deviation of duplicates from one representative experiment of two. For endpoint titers, only means are shown.
(TIF)

Figure S2 **Determination of % anti-FL Abs in sequential serum samples from dengue patients by capture ELISA using DENV1 and DENV2 mutant VLPs.** (A, B) Serial dilutions of sera (#237 6 and 18 months post-infection) were subjected to a capture ELISA using DENV1 WT and mutant VLPs containing mutations in FL (W101A+F108A) (A) and DENV2 WT and mutant VLPs (W101A+F108A) (B). The data are presented as in Figure 2. (C) The % anti-FL Abs in sera of another 3 patients (#194, #256 and #274) determined by capture ELISA using DENV1 and DENV2 WT and mutant VLPs. Data are means with standard deviation of duplicates from one representative experiment of two. For endpoint titers, only means are shown.
(TIF)

Figure S3 **Relationship of [anti-E Abs], % anti-FL Abs and [anti-FL Abs] determined by virion- and VLP-capture ELISA of DENV1 versus DENV3 or DENV2 systems.** (A, B, C) [anti-E Abs] (A), % anti-FL Abs (B) and [anti-FL Abs] (C) in serum samples from 14 DENV3 cases were determined by DENV3 virion- and VLP-capture ELISA and were plotted against those determined by DENV1 virion- and VLP-capture ELISA. (D, E, F) [anti-E Abs] (D), % anti-FL Abs (E) and [anti-FL Abs] (F) in serum samples from 7 DENV2 cases were determined by DENV2 virion- and VLP-capture ELISA and were plotted against those determined by DENV1 virion- and VLP-capture ELISA. DENV3 (H87 strain) and DENV2 (NGC strain) virions were used in the virion-capture ELISA. DENV3 and DENV2 prM/E plasmids producing WT and FL mutant (W101A+F108A) VLPs were used to determine the % anti-FL Abs [24]. Data are means with standard deviation of duplicates from one representative experiment of two.
(TIF)

Table S1 **Concentration of total anti-E Abs and anti-FL Abs and proportion of anti-FL Abs in sera of 26 dengue cases 12 months post-infection.**
(DOC)
