# Supplementary figures and images for: Analysis of Cross-Reactive Antibodies Recognizing the Fusion Loop of Envelope Protein and Correlation with Neutralizing Antibody Titers in Nicaraguan Dengue Cases
Source: PLoS Negl Trop Dis. 2013 Sep 19;7(9):e2451. doi: 10.1371/journal.pntd.0002451 (PMC3777924; doi:10.1371/journal.pntd.0002451)

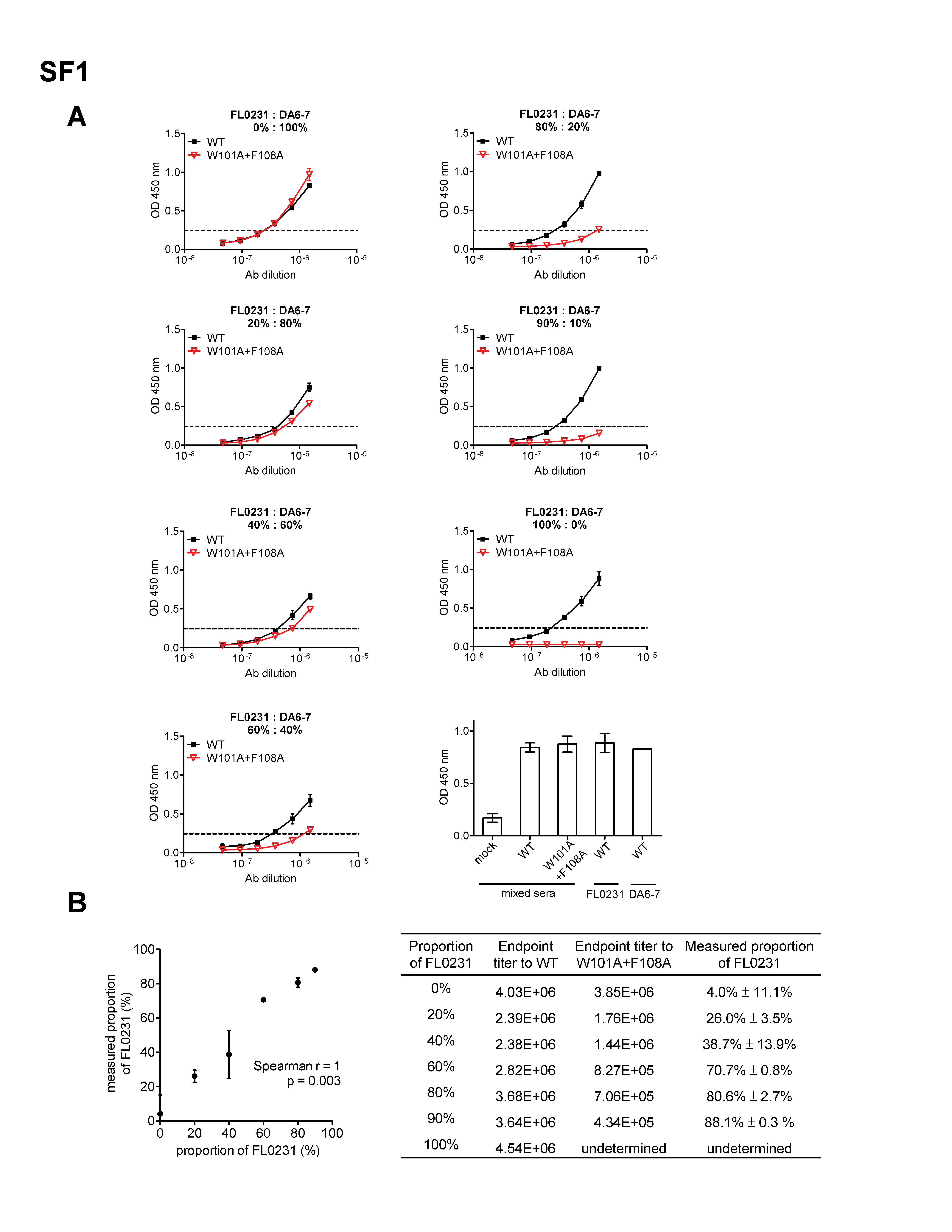

Supplement: Figure S1 — Determination of % anti-FL Abs in mixtures of mAbs containing different proportions of an anti-FL mAb by VLP-capture ELISA. (A) Increasing amounts of mouse mAb FL0231, which recognizes the FL, was mixed with mouse mAb DA6-7, which recognizes E domain III, such that the proportion of anti-FL mAb increased from 0% to 100%. Different mixtures were subjected to a capture ELISA using DENV1 WT and mutant VLPs containing mutations in the FL epitope (W101A+F108A). The bar graph displaying results of an anti-E ELISA shows that comparable amounts of WT and mutant VLPs were added based on recognition of E by pooled human dengue-immune sera. (B) A linear relationship between the proportion of FL0231 (anti-FL mAb) added and the measured proportion of FL0231 was noted (P = 0.003, two-tailed Spearman correlation test). % anti-FL Abs (measured proportion of FL0231) = [1 – endpoint titer to mutant VLPs/endpoint titer to WT VLPs]×100%. Data are means with standard deviation of duplicates from one representative experiment of two. For endpoint titers, only means are shown. (TIF) [file pntd.0002451.s001.tif]

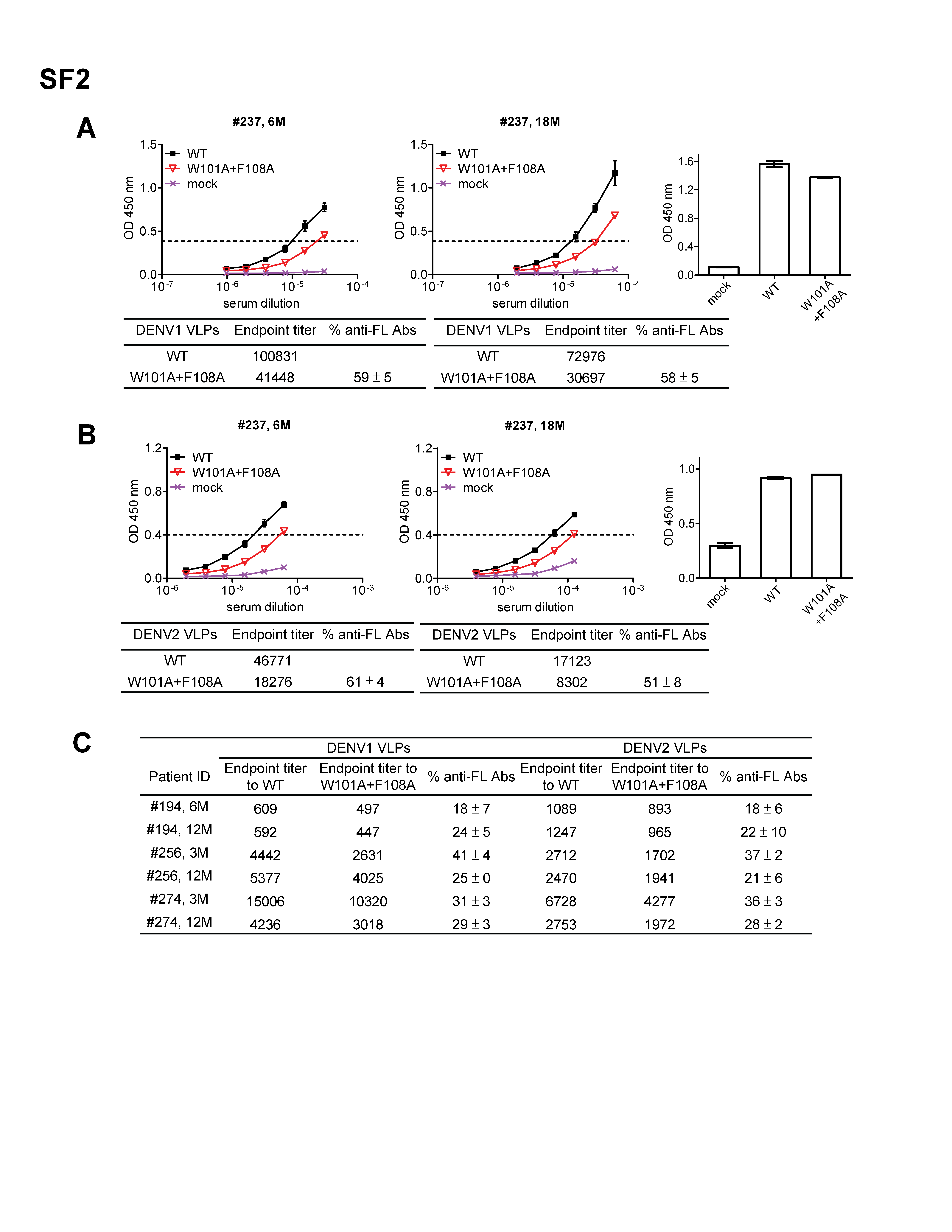

Supplement: Figure S2 — Determination of % anti-FL Abs in sequential serum samples from dengue patients by capture ELISA using DENV1 and DENV2 mutant VLPs. (A, B) Serial dilutions of sera (#237 6 and 18 months post-infection) were subjected to a capture ELISA using DENV1 WT and mutant VLPs containing mutations in FL (W101A+F108A) (A) and DENV2 WT and mutant VLPs (W101A+F108A) (B). The data are presented as in Figure 2. (C) The % anti-FL Abs in sera of another 3 patients (#194, #256 and #274) determined by capture ELISA using DENV1 and DENV2 WT and mutant VLPs. Data are means with standard deviation of duplicates from one representative experiment of two. For endpoint titers, only means are shown. (TIF) [file pntd.0002451.s002.tif]
