# Supplementary material for: Analysis of Cross-Reactive Antibodies Recognizing the Fusion Loop of Envelope Protein and Correlation with Neutralizing Antibody Titers in Nicaraguan Dengue Cases
Source: PLoS Negl Trop Dis. 2013 Sep 19;7(9):e2451. doi: 10.1371/journal.pntd.0002451 (PMC3777924; doi:10.1371/journal.pntd.0002451)
Supplement: Table S1 — Concentration of total anti-E Abs and anti-FL Abs and proportion of anti-FL Abs in sera of 26 dengue cases 12 months post-infection. (DOC) [file pntd.0002451.s004.doc]

**Table S1. Concentration of total anti-E Abs and anti-FL Abs and proportion of anti-FL Abs in sera of 26 dengue cases 12 months post-infection**

| Patient ID | Immune status^a^ | Current infecting serotype^b^ | [anti-E Abs]  (μg/ml)^c^ | % anti-FL Abs  (%)^c^ | [anti-FL Abs]  (μg/ml)^c^ |
| --- | --- | --- | --- | --- | --- |
| 133 | secondary | D2 | 179.1 ± 21.4 | 38 ± 2 | 68.1 |
| 295 | secondary | D2 | 118.9 ± 12.7 | 37 ± 0 | 44.0 |
| 296 | secondary | D2 | 163.2 ± 6.5 | 36 ± 1 | 58.8 |
| 299 | secondary | D2 | 370.9 ± 26.4 | 57 ± 1 | 211.4 |
| 300 | secondary | D2 | 145.7 ± 4.4 | 18 ± 0 | 26.2 |
| 301 | secondary | D2 | 240.2 ± 8.9 | 63 ± 1 | 151.3 |
| 303 | secondary | D2 | 306.3 ± 6.3 | 24 ± 3 | 73.5 |
| 305 | secondary | D2 | 224.1 ± 24.7 | 36 ± 3 | 80.7 |
| 306 | secondary | D2 | 71.1 ± 6.8 | 20 ± 1 | 14.2 |
| 309 | secondary | D2 | 438.6 ± 0 | 26 ± 0 | 114.0 |
| 316 | secondary | D2 | 353.9 ± 35.0 | 24 ± 3 | 84.9 |
| 341 | secondary | D2 | 266.8 ± 15.7 | 21 ± 3 | 56.0 |
| 351 | secondary | D3 | 83.5 ± 5.9 | 48 ± 1 | 40.1 |
| 368 | secondary | D3 | 545.4 ± 26.5 | 56 ± 1 | 305.4 |
| 374 | secondary | D3 | 91.9 ± 1.1 | 23 ± 4 | 21.1 |
| 385 | secondary | D3 | 78.3 ± 3.0 | 63 ± 2 | 49.3 |
| 395 | secondary | D3 | 104.5 ± 1.7 | 61 ± 0 | 63.7 |
| 400 | secondary | D3 | 344.2 ± 52.7 | 38 ± 0 | 130.8 |
| 403 | secondary | D3 | 124.2 ± 12.4 | 49 ± 0 | 60.9 |
| 412 | secondary | D3 | 440.9 ± 25.1 | 16 ± 0 | 70.5 |
| 421 | secondary | D3 | 43.3 ± 1.6 | 45 ± 1 | 19.5 |
| 429 | secondary | D3 | 102.5 ± 7.5 | 54 ± 1 | 55.4 |
| 433 | secondary | D3 | 1140.9 ± 71.8 | 19 ± 4 | 216.8 |
| 444 | secondary | D3 | 169.9 ± 7.1 | 6 ± 2 | 10.2 |
| 452 | secondary | D3 | 167.4 ± 5.4 | 19 ± 2 | 31.8 |
| 454 | secondary | D3 | 18.2 ± 0.9 | 16 ± 2 | 2.9 |

^a,b^ Immune status and the current infecting serotype were determined as described in Methods. D=DENV.

^c^ [anti-E Abs], [anti-FL Abs] and % anti-FL Abs were determined as described in Methods.
